# Changes in Lacrimal Punctum Position and Tear Meniscus Height after Correction of Horizontal Laxity in Involutional Lower Eyelid Entropion

**DOI:** 10.1155/2023/4113151

**Published:** 2023-01-17

**Authors:** Tatsuro Yokoyama, Aric Vaidya, Shinjiro Kono, Hirohiko Kakizaki, Yasuhiro Takahashi

**Affiliations:** ^1^Department of Oculoplastic, Orbital & Lacrimal Surgery, Aichi Medical University Hospital, Aichi, Japan; ^2^Department of Oculoplastic, Orbital & Lacrimal Surgery, Kirtipur Eye Hospital, Kathmandu, Nepal

## Abstract

**Purpose:**

To examine changes in the position of the lower eyelid punctum and tear meniscus height (TMH) after correction of horizontal laxity of the lower eyelid in involutional lower eyelid entropion.

**Methods:**

This prospective, observational study included 42 sides of 36 patients with involutional entropion who underwent a lateral tarsal strip procedure or transcanthal canthopexy (+ lower eyelid retractor advancement). The horizontal distance from the medial margin of the lower lacrimal punctum to the medial canthus was measured using ImageJ software. TMH was measured using anterior segment optical coherence tomography. All measurements were performed preoperatively, at postoperative 3-month and at postoperative 6-month.

**Results:**

The lower lacrimal punctum significantly shifted laterally at 3-month follow-up and slightly returned toward its original position at 6-month follow-up (Friedman's test, *P*  <  0.001). Although the differences did not reach statistical significance, TMH in the lower eyelid increased at 3-month follow-up and then slightly decreased at 6-month follow-up (Friedman's test, *P* = 0.076).

**Conclusions:**

The results of this study imply that lateral shift of the lower lacrimal punctum prevents effective drainage of tears accumulated in the lacrimal lake, resulting in increased TMH after correction of horizontal laxity of the lower eyelid in involutional entropion.

## 1. Introduction

Involutional lower eyelid entropion (involutional entropion) is the most common type of entropion in which the lower eyelid turns inwardly [1]. Corneal abrasion by the cilia causes ocular pain, irritation, itching, burning sensation, tearing, photophobia, conjunctival injection, discharge, and vision loss [1–3].

The main etiologic factors for the development of involutional entropion are the vertical and horizontal laxities of the lower eyelid [1]. For addressing the vertical laxity, the lower eyelid retractor (LER) is advanced, while the horizontal laxity is corrected by horizontal tightening or shortening procedures of the lower eyelid, such as the lateral tarsal strip (LTS) procedure, transcanthal canthopexy (TCC), and wedge resection [1].

When the lower eyelid is horizontally tightened or shortened, the lower lacrimal punctum is shifted laterally [4]. A laterally shifted lower lacrimal punctum is away from the lacrimal lake, causing interruption of effective tear drainage. On the contrary, surgical correction of involutional entropion decreases reflex tear secretion and simultaneously corrects lower lacrimal punctum malposition. In addition, horizontal tightening/shortening may improve the lacrimal pump function [5–7]. None of the previous studies demonstrated postoperative changes in lacrimal punctum position and tear meniscus height (TMH) after surgical correction of the horizontal laxity in patients with involutional entropion.

In this study, we examined changes in the horizontal position of the lower lacrimal punctum and TMH after LTS or TCC for addressing the horizontal laxity in patients with involutional entropion.

## 2. Methods

### 2.1. Ethics Approval

The Institutional Review Board (IRB) of Aichi Medical University Hospital approved this study, which was conducted in accordance with the tenets of the Declaration of Helsinki and its later amendments (approval number, 2022-172). The IRB granted a waiver of informed consent for this study based on the ethical guidelines for medical and health research involving human subjects established by the Japanese Ministry of Education, Culture, Sports, Science, and Technology and by the Ministry of Health, Labour, and Welfare. The waiver was granted because the study was not an interventional study. Nevertheless, at the request of the IRB, an outline of the study was published on the Aichi Medical University website that was available for public viewing, which also gave the patients the option to refuse to participate in the study, although none did. Personal identifiers were removed from the records prior to data analysis.

### 2.2. Study Design and Patients

This prospective, observational study included Japanese patients in whom involutional lower eyelid entropion was corrected by one of the authors (YT) from October 2017 to January 2022. Patients with a previous history of eyelid or lacrimal surgery were excluded from this study. A patent lacrimal drainage system was confirmed before surgery in all patients.

### 2.3. Data Collection

The following data were collected: age, sex, affected side, results of the pinch test, and surgical procedures. The horizontal positions of the lower lacrimal punctum and TMH were measured preoperatively, at postoperative 3 months, and at postoperative 6 months.

**Figure 1:**
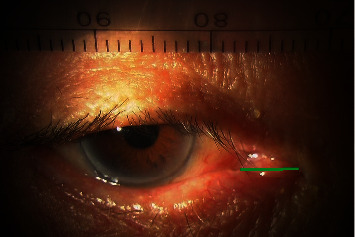
Measurement of the horizontal position of the lower lacrimal punctum. After exposure of the lower punctum, the horizontal distance from the medial margin of the lower lacrimal punctum to the medial canthus (green solid line) was measured.

To measure the horizontal position of the lacrimal punctum in the lower eyelid, slit-lamp photos were taken with the patients' heads and chins fixed and the eyes in the primary position. To avoid changes in the lower eyelid position vertically and horizontally, we gently turned the eyelid margin by a finger to expose the lower lacrimal punctum. A ruler was contained in the photos. The photos were captured, and the horizontal distance from the medial margin of the lacrimal punctum (white zone boundary [8]) to the medial canthus was measured using ImageJ software (National Institute of Health, Bethesda, MD) (Figure 1). The measurements were made by converting pixel numbers to distance based on the ruler contained in the same photo.

TMH was measured on the sagittal plane through the centers of the upper and lower eyelids using an anterior segment optical coherence tomography (CASIA2, TOMEY Corporation, Aichi, Japan). Before surgery, an inverted lower eyelid was returned to the normal eyelid position by a digital push of the lower eyelid in the inferior direction. Patients were directed to blink several times, then stop blinking, and keep their eyes open to prevent another inward turn of the lower eyelid [9]. At that time, the TMH was measured. Postoperatively, the TMH was measured after blinking several times.

### 2.4. Surgical Procedure

The details of each surgical procedure are presented in our previous studies [10, 11].

#### 2.4.1. LER Advancement

Under local anesthesia, the skin was incised 3 mm below the cilia. The layer under the orbicularis oculi muscle (OOM) was dissected toward the cilia. The anterior layer of the LER on the tarsus was detached inferiorly until the lower margin of the tarsus was exposed. The posterior layer of the LER was detached from the conjunctiva. The orbital septum was incised transversely just below the junction between the anterior layer of the LER and the orbital septum. The OOM in the eyelid margin was slightly debulked to facilitate outward rotation of the eyelid margin. Then, a site 2 mm below the edge of the posterior layer was fixed to the lower edge of the tarsus using a 6-0 Asflex® (Kono Seisakusho Co., Ltd., Tokyo, Japan) suture with simultaneous advancement of the anterior layer to reinforce the posterior layer. We added 2 additional sutures, and then the pretarsal OOM and the lower edge of the tarsus were secured at three points. Finally, the skin was sutured with 6-0 Asflex® sutures.

#### 2.4.2. LTS

Under local anesthesia, a 10 mm skin incision was made along the lateral canthal rhytids. A lateral canthotomy was performed, and the inferior crus of the lateral canthal band and the Lockwood ligament were severed. A 7 mm incision through the conjunctiva and LERs was made immediately below the tarsus in the temporal portion of the eyelid to free the temporal tarsal attachment. The anterior and posterior lamellae of the temporal portion of the eyelid were separated. The skin and conjunctiva at the mucocutaneous junction were trimmed, and the conjunctiva on the tarsus was removed to fashion the tarsal strip. The tarsal strip was shortened by the appropriate amount and then fixed to the inside of the lateral orbital wall with a 5-0 Prolene® (Ethicon Inc., Bridgewater, NJ) suture. After a lateral canthoplasty, the OOM and skin were sutured with 6-0 Asflex® sutures.

#### 2.4.3. TCC

Under local anesthesia, a 6 mm skin incision was made along the lateral canthal rhytids, just anterior to the lateral orbital rim. A stab incision was made at the lower eyelid margin, just medial to the commissure. Needles with a double-armed 5-0 Prolene® suture were inserted into the stab incision, passed through the hard lateral retinaculum or periosteum, and pulled out from the skin incision area. After a firm ligation of this suture, the skin was closed with 6-0 Asflex® sutures.

### 2.5. Statistical Analyses

Patient age and measurement results are expressed as the mean value ± standard deviation. The position of the lower lacrimal punctum and TMH were compared among 3 measurement periods using Friedman's test and Bonferroni correction because some of the measurement results did not have a normal distribution. In addition, the horizontal position of the lower lacrimal punctum and TMH were compared between patients who underwent LTS and TCC using the Mann–Whitney *U* test. Preoperative TMH was compared between the affected and unaffected sides in patients with unilateral involutional entropion using Student's *t*-test. All statistical analyses were performed using SPSS™ ver. 26 software (IBM Japan, Tokyo, Japan). A *P* value of <0.050 was considered statistically significant.

We also performed a post hoc analysis of the validity of the sample size in this study. The effect size was calculated based on the mean values and standard deviations of TMH in the lower eyelid. The *α* error was set as 0.05, and the power of the test (=1 − *β* error) was calculated using G*∗*Power software version 3.1.9.7 (Heinrich-Heine-Universität Düsseldorf, Düsseldorf, Germany).

## 3. Results

Data on demography and measurement results are shown in Table 1. This study included 42 sides from 36 patients. Bilateral involutional entropion was seen in 6 patients. The pinch test was positive (8 mm anterior displacement from the globe) before surgery on 36 sides. LTS and TCC were performed on 15 and 27 sides, respectively. None of the patients had any disease affecting wound healing or took medications, which could also affect the recovery of the surgical site. The power of the test (=1 − *β* error) was 1.000, indicating the validity of the sample size in this study.

The results of the statistical comparison are shown in Tables 1to 4. The horizontal distance from the lower punctum to the medial canthus got longer at the 3-month follow-up period. At the 6-month follow-up period, the distance got shorter from the baseline measured at 3-month follow-up, but it was still longer than that measured preoperatively. The distance measured at 3-month follow-up was significantly longer than that measured preoperatively (*P* = 0.035). The difference in the distances measured preoperatively and at 6-month follow-up did not reach statistical significance (*P* = 0.194). Similarly, the distances measured at 3- and 6-month follow-up were also not significantly different (*P* = 1.000).

The TMH in the upper eyelid did not change significantly after surgery (*P* = 0.801). TMH in the lower eyelid increased once at the 3-month follow-up period and then slightly decreased at the 6-month follow-up period. But these changes did not reach statistical significance (*P* = 0.076).

The lower punctum position and TMH measured at each measurement period were not different between patients who underwent LTS and TCC (*P*  >  0.050). Preoperative TMH was not different between the affected and unaffected sides in patients with unilateral involutional entropion (*P*  >  0.050).

Involutional entropion was successfully treated, and none of the patients experienced recurrence until 6-month follow-up. None of the patients had apparent lower eyelid retraction and ectropion after the surgeries performed. Although all patients obtained horizontal tightness after surgery once, 4 patients showed a positive pinch test at the 6-month follow-up period (after LTS in 2 cases and after TCC in 2 cases).

## 4. Discussion

This study examined changes in the position of the lower lacrimal punctum and TMH after correction of horizontal laxity of the lower eyelid in patients with involutional entropion. We found out that the lower lacrimal punctum shifted laterally significantly. Although the differences did not reach statistical significance, TMH in the lower eyelid increased after the surgeries. The lacrimal lake is located adjacent to the lacrimal caruncle and is vertically aligned with the lacrimal papilla [12]. A laterally shifted lower lacrimal punctum is, therefore, away from the lacrimal lake, which may interfere with the effective drainage of tears accumulated in the lacrimal lake. This may be reflected in the measurement results of this study.

Preoperative TMH was not different between the affected and unaffected sides in patients with unilateral involutional entropion. Although involutional entropion can cause reflex tear secretion by cilia-induced ocular surface abrasion [3], decreased corneal sensitivity in old patients may not increase the amount of reflex tear secretion [13]. In addition, malposition of the lower lacrimal punctum may also not affect the function of tear drainage.

Horizontal tightening/shortening of the lower eyelid may improve the function of the lacrimal pump [5–7]. However, TMH tended to increase after LTS or TCC in this study. One of the possible reasons was that horizontal tightening/shortening does not always cure lacrimal pump failure. Previous studies showed that 41.2–87% of patients experienced complete or nearly complete relief of epiphora after horizontal tightening/shortening [5–7]. Another possible reason was that the degree of TMH elevation by the lateral shift of the lower lacrimal punctum surpassed the degree of TMH reduction by the effectiveness of horizontal tightening/shortening.

Although the differences did not reach statistical significance, the horizontal distance between the lower lacrimal punctum and the medial canthus and TMH in the lower eyelid measured at 6-month follow-up decreased slightly from the baseline measured at 3-month follow-up. Since the scar gradually softens during the scar remodeling phase within a several weeks to a few years [14, 15], this may be caused by mild improvement of cicatricial contracture in the lateral canthus.

We supposed that LTS secures a tighter fixation, resulting in a more lateral shift of the lower lacrimal punctum and no return of the lower punctum position at 6-month follow-up, compared to TCC. However, the measurement results were not different between patients who underwent LTS and TCC. Our previous reports showed no recurrence of involutional entropion after both LER advancement + LTS and LER advancement + TCC [10, 11]. These imply that even TCC provides a horizontal tightness equivalent to LTS.

In LTS and TCC, the lower eyelid is drawn laterally in one direction. On the contrary, in the Kuhnt–Szymanowski procedure [16], the lower eyelid is drawn both medially and laterally [4]. This may induce a less lateral shift of the lower lacrimal punctum [4]. Although we did not evaluate medial and lateral canthal laxities using lateral and medial distraction tests, the Kuhnt–Szymanowski procedure may be a better option to prevent excess lateral shift of the lower lacrimal punctum and following epiphora, particularly in patients with severe laxity of the medial canthus.

There are a few limitations to this study. First, this study included only Japanese patients. Since there are known racial differences in eyelid anatomy [17], the results may not be applicable to other races. Second, all the measurements were performed by a single examiner, which could affect the reliability of this study. Third, quantification of the results of the pinch test may provide more information for this study.

In conclusion, the lower lacrimal punctum shifted laterally, and TMH in the lower eyelid tended to increase after correcting horizontal laxity of the lower eyelid in patients with involutional entropion. These results indicate that a laterally shifted lower lacrimal punctum was unable to effectively drain tears accumulated in the lacrimal lake, resulting in increased TMH after surgery.

## Figures and Tables

**Table 1:** Demographic data and results of measurements and statistical comparison.

Patient number/sides	36/42

M/F	18/18
R/L	19/23
Patient age (years)	75.9 ± 8.1
Positive pinch test (sides)	
Preoperative	36
Postoperative 6 months	4
Surgical procedure	
LER advancement + LTS	15
LER advancement + TCC	27
Horizontal position of lower lacrimal punctum (mm)	
Preoperative	3.95 ± 0.24
Postoperative 3 months	4.79 ± 0.23
Postoperative 6 months	4.56 ± 0.23
*P* value	<0.001
TMH (*μ*m) upper eyelid	
Preoperative	196.8 ± 14.9
Postoperative 3 months	195.1 ± 14.3
Postoperative 6 months	194.2 ± 13.6
*P* value	0.801
Lower eyelid	
Preoperative	276.0 ± 29.4
Postoperative 3 months	311.1 ± 29.6
Postoperative 6 months	303.2 ± 25.3
*P* value	0.076

M, male; F, female; R, right; L, left; LER, lower eyelid retractor; LTS, lateral tarsal strip; TCC, transcanthal canthopexy; TMH, tear meniscus height.

**Table 2:** The results of Bonferroni correction.

Lower punctum position	Postoperative 3 months	Postoperative 6 months
Preoperative	0.035	0.194
Postoperative 3 months	—	1.000

**Table 3:** Comparison of measurement results between patients who underwent lateral tarsal strip (LTS) procedure and transcanthal canthopexy (TCC).

	LTS	TCC	*P* value
Horizontal position of lower lacrimal punctum (mm)			
Preoperative	3.57 ± 1.42	4.16 ± 1.64	0.232
Postoperative 3 months	4.81 ± 1.62	4.77 ± 1.42	0.783
Postoperative 6 months	4.36 ± 1.64	4.67 ± 1.40	0.896
TMH (*μ*m)			
Upper eyelid			
Preoperative	173.2 ± 88.2	210.0 ± 100.3	0.242
Postoperative 3 months	205.3 ± 98.1	189.5 ± 90.9	0.646
Postoperative 6 months	172.3 ± 79.3	206.3 ± 92.2	0.372
Lower eyelid			
Preoperative	248.9 ± 199.4	291.0 ± 188.1	0.416
Postoperative 3 months	316.7 ± 224.9	307.9 ± 174.9	0.503
Postoperative 6 months	291.3 ± 151.4	309.9 ± 173.4	0.783

TMH, tear meniscus height.

**Table 4:** Comparison of preoperative tear meniscus height (TMH) between the affected and unaffected sides in patients with unilateral involutional entropion.

	Affected side	Unaffected side	*P* value
Preoperative upper TMH (*μ*m)	202.8 ± 104.7	210.3 ± 81.9	0.759
Preoperative lower TMH (*μ*m)	270.5 ± 203.0	238.7 ± 113.7	0.456

## Data Availability

The data supporting the results of this study are available from the corresponding author upon request.
